# Invasive Predators Deplete Genetic Diversity of Island Lizards

**DOI:** 10.1371/journal.pone.0012061

**Published:** 2010-08-10

**Authors:** Amandine Gasc, M. C. Duryea, Robert M. Cox, Andrew Kern, Ryan Calsbeek

**Affiliations:** Department of Biological Sciences, Dartmouth College, Hanover, New Hampshire, United States of America; Midwestern University, United States of America

## Abstract

Invasive species can dramatically impact natural populations, especially those living on islands. Though numerous examples illustrate the ecological impact of invasive predators, no study has examined the genetic consequences for native populations subject to invasion. Here we capitalize on a natural experiment in which a long-term study of the brown anole lizard (*Anolis sagrei*) was interrupted by rat invasion. An island population that was devastated by rats recovered numerically following rat extermination. However, population genetic analyses at six microsatellite loci suggested a possible loss of genetic diversity due to invasion when compared to an uninvaded island studied over the same time frame. Our results provide partial support for the hypothesis that invasive predators can impact the genetic diversity of resident island populations.

## Introduction

Invasive species are generally recognized as one of the most pervasive threats to natural populations [1]. Their impact can be severe, and is perhaps nowhere more insidious than on islands, where geographic isolation magnifies the long-term consequences of habitat degradation and species loss [1]. Of particular interest to many studies of island biology has been the impact of invasive rats (*Rattus* spp). Invasive rats have colonized more than 80% of the world's islands, and their extremely catholic diets impact plants, invertebrates, mammals, birds, and reptiles [2]. As such, invasive rats are generally thought responsible for causing the majority of extinctions on islands [2].

Whereas the ecological impact of invasive species, including rats, has been well documented [2], no study to date has examined the population genetic consequences of an invasive predator for resident species. It is generally assumed that the genetic impact of invasive species will be to reduce effective population size [3], [4] and depress the standing crop of genetic variation of natural populations. Population genetic theory posits that the first genetic signature of a population crash should be a marked reduction in the number of alleles per locus (i.e., allelic richness) as rare alleles are lost via genetic drift [5]. Given a sufficiently narrow bottleneck, this change in allelic richness should be followed by a concomitant loss of heterozygosity [5]. These two changes can impair adaptation to changing environments and may be considered a loss of evolutionary potential [6], [7]. Such an effect has been shown in populations founded by a small number of individuals [8], [9], and it is a logical extension that invasive predators that depress population sizes might have similar effects.

Although a growing number of studies have demonstrated the genetic consequences for exotic species following invasion [10], [11], including the negative consequences of hybridization between exotic and native species [12], [13], the hypothesis that invasive species should have negative population genetic consequences for *residents* has hitherto gone untested. In part, this may reflect the obvious ethical impediments to performing experimental manipulations with invasive species in nature. Here we capitalize on a natural experiment [1], in which our long-term study of island lizards in The Bahamas was interrupted by rat invasion, to document changes in genetic diversity following a population decline induced by invasive predators. Using microsatellite markers to compare levels of genetic diversity on invaded and uninvaded “control” islands before and after the period of rat invasion, we show that a population bottleneck following rat invasion led to significant loss of allelic richness and a non-significant decrease in population-level heterozygosity. We discuss our results in terms of their implications for conservation biology, especially on remote islands.

## Methods

All research carried out in this study was approved and carried out under IACUC protocol 07-02-03. We have studied natural selection on small (600–2500 m^2^) islands in The Bahamas since 2005 [14]. These small islands are naturally home to anoles and maintain stable populations in the absence of major disturbance [15]. Lizards on all islands are naturally susceptible to predation by birds (e.g., mockingbirds, *Mimus polyglottos*, and green herons, *Butroides striatus*). The islands used in our studies are located within 2 km of each other and are dominated by a mixture of small trees and native shrubs (e.g., sea grape, *Coccoloba uvifera*; buttonwood, *Conocarpus erectus*) that make up the majority of perching habitat used by anoles.

Each spring, we capture *Anolis sagrei* lizards from the large island of Great Exuma, sample a small piece (2 mm) of tail tissue from each individual for genetic analyses, and introduce propagules of ca. 200 lizards to each of several offshore islands which we have previously denuded of resident lizards. Each fall, ca. 4 months following introduction, we recapture all surviving lizards (mean survival: 32%, range  = 29.9–40.5%) and remove them from the islands. During 2008, our fall census on one experimental island turned up a single surviving male lizard, and inspection of the island's vegetation showed clear signs of herbivory by rodents. Two independent observations of rats on the island (RC and RMC) confirmed invasion. Following our fall censuses, we exterminated the rats using standard poisoning protocols [16]. Rat poison was administered when no lizards were present on the islands and poison dispensers were empty several months prior to lizard emergence. Thus, we can be sure that no lizard consumed rat poison.

The spring following rat extermination, the lizard population on the invaded island showed signs of initial recovery, presumably from eggs laid prior to rat predation. We captured all (N = 13) lizards present on this island and sampled an equal number of lizards from a second study island that was not affected by rat invasion and which therefore served as a control.

We genotyped tissue samples from both islands using a library of six polymorphic microsatellite markers (AAGG-38, AAAG-70, AAAG-76, AAG-91, AAAG-94, and AAAG-95) and amplification conditions modified from [17] (Appendix S1). We performed electrophoresis on an ABI 3100 automated capillary DNA sequencer, and sized alleles against the GeneScan-500 LIZ size standard using Genemapper v3.5 (Applied Biosystems). We tested for linkage disequilibrium and heterozygote deficiency using GenePop (v4.0.1)[18]. To standardize our sampling effort, especially for allelic richness measures, we genotyped an equal number of randomly selected tissue samples collected from each island prior to invasion. We calculated allelic richness and average heterozygosity using Arlequin v.2.0 [19], and we compared these values between treatments using ANOVA, with invasion treatment (invaded vs. uninvaded) as a factor, considering the genetic locus as independent units of observation.

To quantify the effect of rat invasion on the effective population size of *A. sagrei*, we used approximate Bayesian computation (ABC, e.g. [20]) and estimated the population effective mutation parameter with a regression step after simulation using the methods of Beaumont et al. [21]. We performed coalescent simulations under the standard neutral model (SNM) with a stepwise mutational model to simulate the evolution of microsatellites [20], [22]. Five million simulations were performed for each population sample, conditional on the number of individuals genotyped. We then summarized each simulation replicate by the mean and variance in the number of alleles observed to obtain ABC estimates of theta ( = 4 Nμ), the population effective mutation rate.

## Results

We detected no significant linkage disequilibrium among pairs of loci used in our study and we conclude that the six loci are inherited independently. We detected no evidence for the presence of null alleles, nor did we find any systematic deficits in the numbers of heterozygotes across loci in our source populations (but see below for variation among sites).

**Figure 1:**
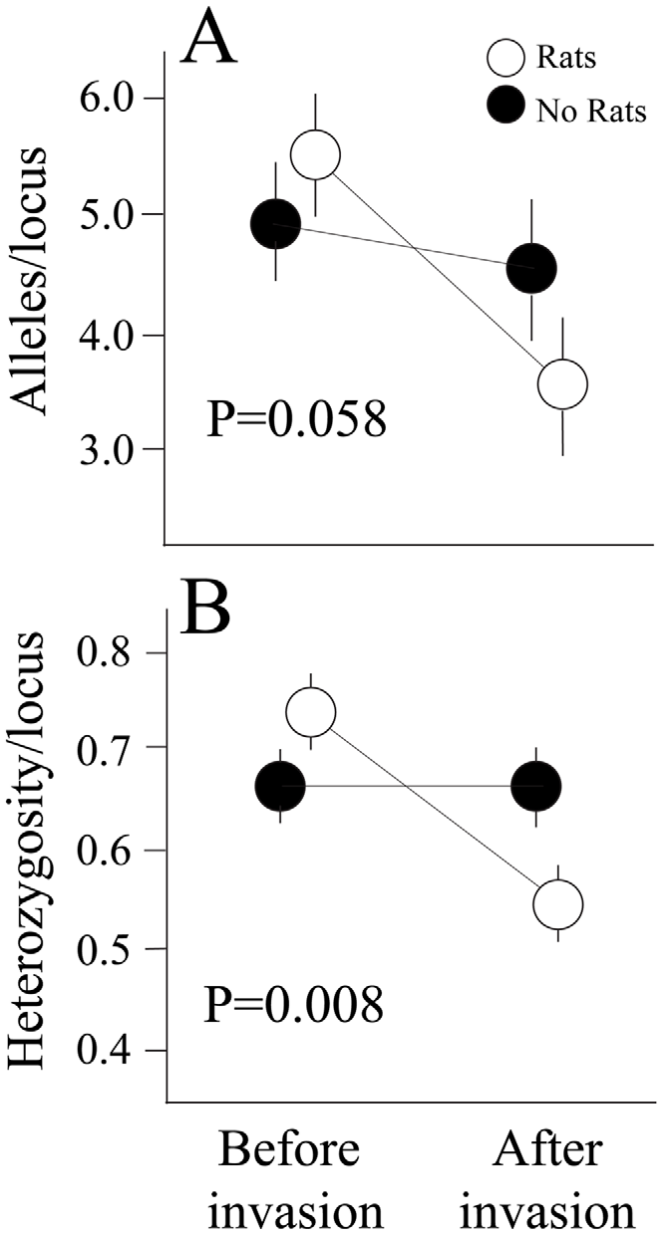
Changes in genetic diversity following rat invasion. The lizard population that experienced rat invasion showed significant loss of A. allelic richness and B. heterozygosity compared to the uninvaded “control”. Analyses show means (+SE) across six microsatellite loci. Significance values are from repeated measures ANOVA.

**Figure 2:**
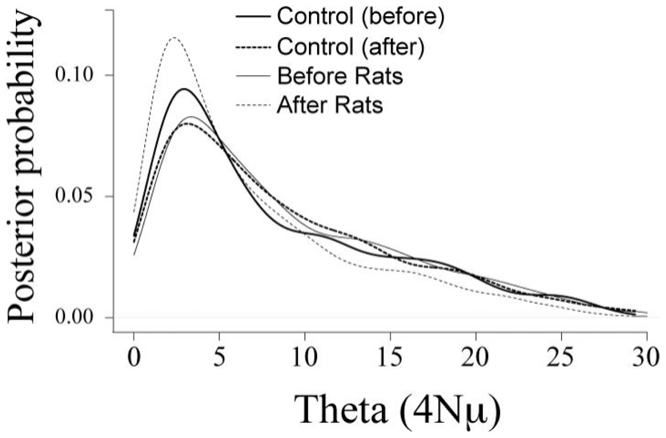
Approximate Bayesian estimation of theta. Analyses revealed no measurable change in effective population size following rat invasion.

As predicted, the lizard population that had been invaded by rats showed reduced levels of allelic richness compared to our control population, though this result was not significant probably owing in part to low statistical power (i.e., 13 lizards genotyped at six loci; Repeated Measures ANOVA F_1,10_ = 4.58 P = 0.058; Fig. 1A). The change in allelic richness within the invaded population was significantly greater than zero (one sample t-test; t = 4.71, P<0.01). Allelic richness in control populations also decreased over the time scale of our study (Fig. 1A), but this small change in the mean was not statistically different from zero (one sample t-test t = 0.74, P = 0.74). Heterozygosity levels decreased significantly following rat invasion (Repeated Measures ANOVA F_1,10_ = 10.72 P = 0.008; Fig. 1B). As the coalescent process predicts a large stochastic variance surrounding any point estimate of heterozygosity, we decided to perform coalescent estimation of the neutral mutation parameter to determine if the observed differences were significant. Simulations based on the coalescent showed no differences in effective population size following rat invasion (Fig. 2). This indicates that while the point estimates are suggestive, they may not be beyond the variation one would expect under the standard neutral model, especially given the fact there had yet to be a reproductive generation of lizards on the island.

## Discussion

Invasive species pose one of the greatest threats to the planet's biodiversity [23], [24]. Despite the widely-recognized potential for invasive species to negatively impact the ecology and population biology of resident species [25], little has been done to quantify the impact that invasive species may have on the evolutionary potential of these populations. We have provided some preliminary support for the hypothesis that invasive predators can deplete the population genetic diversity of a natural resident species. Despite our rapid response to invasion, and elimination of the invasive rats (within one lizard generation), the native lizard population appears to have suffered a reduced evolutionary potential in terms of the loss of genetic diversity.

Although our results suggest a possible loss of diversity at six neutrally evolving loci, the effects we show likely demonstrate a concomitant loss of diversity in other genes, including some that may be important to the adaptive potential of this species [26]. The impact that invasive predators will have on the genetic diversity of other native species will depend on the particular ecological interactions between native and invasive species, and we point out that our results probably represent a lower bound to the potential loss of genetic diversity given that we eradicated the rats over such a short time scale following invasion. The most conservative analytical approach using Bayesian approximations of the null distribution did not resolve the same differences that we measured with point estimates (Fig. 1). That is, while a parametric hypothesis test points to possible differences in levels of diversity between groups, estimation of population size via the coalescent does not show such an effect. Thus while we see a short term effect, not surprisingly the long term historical effective population size of the species is yet to feel the effects of the ecological invasion. This is because effective population size as estimated from genetic data is the interaction, over many generations, of mutation, selection, and genetic drift. We suggest that, had we not responded to the invasion, consequences could have been more severe for local populations, perhaps resulting in local extinctions on these islands.

Natural processes like hurricanes can have similar effects on island populations of anoles in The Bahamas. Previous studies have shown that, following disturbance by hurricanes, extinguished anole populations can recover rapidly, either from eggs laid prior to the storm [27], or by over-water dispersal from adjacent islands [28], [29]. Others have documented rapid population expansion following island colonization by a small number of individual anoles (e.g., as few as seven; [30]). Rapid recovery following a population bottleneck may mitigate the net loss of genetic diversity [5], [31], and can influence the probability that natural populations retain genetic diversity following invasion. *Anolis* lizards are themselves a noteworthy invasive species in many areas of the globe [32], [33]. Thus, it is likely that even had we not intervened immediately, lizard populations would have recovered of their own accord. However, many island species affected by invasive predators may have a more limited expansion capacity, and a loss of genetic diversity would further reduce their potential to recover from invasions.

Our results provide partial support for a long-standing but previously untested hypothesis, that invasive predators can impact the genetic diversity of native island species. The changes in allelic diversity and the significant loss of heterozygosity that we observed (Fig. 1) are especially noteworthy given that our intervention occurred within a single generation of invasion. Given that brown anoles are excellent colonizers and our study islands are located in close proximity to a large source population, the long-term effects of rat invasion on our study islands are presumably small. However, for species with limited dispersal capabilities, or for those living on remote islands [34], these results should serve as a warning of the potential genetic consequences that invasive species can have on native insular populations.

## Supporting Information

Appendix S1 Amplification conditions modified from Bardeleben et al. (2004). We performed 10 µL PCR reactions with 1 µL template DNA, 1X GeneAmp PCR Buffer II (Applied Biosystems), 1.5 or 2.0 mM MgCl2 (see below), 0.4 mM dNTPs, 0.25 µM of each primer (forward and reverse), and 0.3 U of Taq polymerase. PCR cycles consisted of an initial denaturation step at 94°C for 5 min followed by 29 or 35 cycles (below) of 45 sec at 94°C, 1 min at primer-specific annealing temperatures (Ta, below), and 1 min at 72°C, followed by a final extension for 5 min at 72°C. All PCRs were performed on a DNAEngine Thermal Cycler (Bio Rad).
(0.04 MB DOC)
