# Supplementary material for: Invasive Predators Deplete Genetic Diversity of Island Lizards
Source: PLoS One. 2010 Aug 10;5(8):e12061. doi: 10.1371/journal.pone.0012061 (PMC2919386; doi:10.1371/journal.pone.0012061)
Supplement: Appendix S1 — Amplification conditions modified from Bardeleben et al. (2004). We performed 10 µL PCR reactions with 1 µL template DNA, 1X GeneAmp PCR Buffer II (Applied Biosystems), 1.5 or 2.0 mM MgCl2 (see below), 0.4 mM dNTPs, 0.25 µM of each primer (forward and reverse), and 0.3 U of Taq polymerase. PCR cycles consisted of an initial denaturation step at 94°C for 5 min followed by 29 or 35 cycles (below) of 45 sec at 94°C, 1 min at primer-specific annealing temperatures (Ta, below), and 1 min at 72°C, followed by a final extension for 5 min at 72°C. All PCRs were performed on a DNAEngine Thermal Cycler (Bio Rad). (0.04 MB DOC) [file pone.0012061.s001.doc]

**Appendix S1**: Amplification conditions modified from Bardeleben et al. (2004). We performed 10 µL PCR reactions with 1 µL template DNA, 1X GeneAmp PCR Buffer II (Applied Biosystems), 1.5 or 2.0 mM MgCl­_2_ (see below), 0.4 mM dNTPs, 0.25 µM of each primer (forward and reverse), and 0.3 U of *Taq* polymerase. PCR cycles consisted of an initial denaturation step at 94°C for 5 min followed by 29 or 35 cycles (below) of 45 sec at 94°C, 1 min at primer-specific annealing temperatures (T_a_, below), and 1 min at 72°C, followed by a final extension for 5 min at 72° C. All PCRs were performed on a DNAEngine Thermal Cycler (Bio Rad).

|  | **Locus** | **T_a_ (°C)** | **Mg^2+^ (mM)** | **Number of Cycles** |
| --- | --- | --- | --- | --- |
| 1 | AAGG-38 | 44 | 1.5 | 35 |
| 2 | AAAG-70 | 54 | 1.5 | 29 |
| 3 | AAAG-76 | 54 | 1.5 | 35 |
| 4 | AAAG-91 | 54 | 1.5 | 35 |
| 5 | AAAG-94 | 55 | 2.0 | 35 |
| 6 | AAAG-95 | 46 | 2.0 | 35 |
